# How Customers Evaluate Genitalia versus Torso Sex Toys on Amazon.com: A Content Analysis of Product Reviews

**DOI:** 10.3390/ejihpe12060042

**Published:** 2022-06-01

**Authors:** Nicola Döring, Veronika Mikhailova, Pari-Gole Noorishad

**Affiliations:** 1 Faculty of Economic Sciences and Media, Institute of Media and Communication Science, Technische Universität Ilmenau, 98693 Ilmenau, Germany; veronika.mikhailova@tu-ilmenau.de; 2 School of Psychology, Faculty of Social Sciences, University of Ottawa, Ottawa, ON K1N 6N5, Canada; pnoor033@uottawa.ca

**Keywords:** sex toy, vibrator, masturbator, sex doll, male torso toy, female torso toy, cybersexuality, online product review, online content analysis, sexual script theory

## Abstract

Sex toys are widely marketed on the Internet. Browsing for, buying, and reviewing sex toys online are popular cybersexual activities. The aim of this study was to investigate consumers’ experiences with different types of realistic sex toys via online product reviews on Amazon.com. Toys were categorized in a 2 × 2 design regarding their representation of the human body (genitalia sex toys representing reproductive organs only versus torso toys representing larger parts of the human body) and their depiction of gender (toys representing female versus male body parts). Informed by feminist discourses on sex toys as well as sexual script theory and consumer research, the study explored the overall evaluations (RQ1), most frequently addressed characteristics (RQ2), usage patterns (RQ3), and perceived effects (RQ4) of the four groups of sex toys. A quantitative manual content analysis of *N* = 778 online sex toy reviews showed that 79% of consumers gave popular realistic sex toys positive ratings (RQ1). The most frequently mentioned characteristics were quality, material, and shape (RQ2). Most reviewers were men and used sex toys for solo sexual activities (RQ3). An additional qualitative analysis of *n* = 69 reviews addressing the perceived effects of sex toy use revealed that consumers predominantly mentioned positive effects (RQ4). Genitalia sex toys received better evaluations than torso sex toys and were perceived to be complementary tools to enhance sexual arousal, whereas the use of torso toys entailed anthropomorphization and symbolic social interactions. Implications for future research and design of different types of sex toys are discussed.

## 1. Introduction

Once considered a taboo, sex toys of various forms and shapes are nowadays mass-produced, openly advertised, and vastly available in online shops [1,2]. Sex toys are defined as material objects used to generate or enhance sexual arousal and pleasure in both solo and partnered sex [3]. They come in a plethora of options and different designs, ranging from mainstream battery-operated vibrators [4] to technologically advanced remote-controlled smart devices [5]. Browsing for sex toys online has become a popular cybersexual activity with about 50% of young women and men in different Western countries reporting this behavior [6] and about one-third having used the Internet to purchase a sex toy [7]. Convenience, the possibility of a private and discreet shopping experience without societal judgment, and the opportunity to familiarize oneself with previous experiences of other users via online product reviews have been identified as the main motivating factors for purchasing sex toys online instead of in-person [7,8].

As a result of the benefits of buying sex toys online, the use of sex toys is ramping in Western countries. Recent studies show that 63% of heterosexual men and 65% of heterosexual women report intimate product use both during solo and partnered sexual activities [9]. Despite such wide prevalence, research on sex toys is relatively sparse, particularly in the context of consumer experiences. However, sex toy users themselves make these experiences visible and easily accessible to the broader public and the scientific community by writing online product reviews.

Amazon.com is the largest e-commerce platform in the Western world. It also sells sex toys and allows consumers not only to search for and buy products but also to read and publish online product reviews. Researchers often collect information on online product reviews via Amazon due to its popularity and predominance in the online market [10]. Amazon’s *Sexual Wellness Products* section includes several thousand sex toys of different types and customer reviews on almost all these products. Thus, the aim of the present study is to explore customers’ experiences with popular sex toys based on the online product reviews published on Amazon.com.

### 1.1. Online Sex Toy Reviews

Online product reviews are “peer-generated product evaluations posted on company or third-party websites” [11]. Consumers’ reviews usually consist of a star rating ranging from 1 to 5 stars and a written comment about the product [11]. Online reviews can be considered as electronic word-of-mouth (eWOM) because of their vast availability, ease of access, and interactive nature [12]. Many researchers agree that eWOM plays an important role in customers’ attitudes toward products (e.g., [13,14,15] Given that online reviews are written by those who have used the products, people perceive them as credible sources and tend to trust online reviews more than the general product information provided by the vendors themselves [16,17]. However, it is important to highlight that only a small portion of customers leave product reviews. Usually, customers who were exceptionally satisfied or unsatisfied are more prone to writing a product review [15]. Hence, both very positive and very negative experiences seem to be overrepresented, while more balanced experiences might be underrepresented in product reviews, even though consumers consider balanced reviews the most helpful and credible [11].

Online sex toy reviews can be helpful in exploring users’ experiences with various sex toys. First, the star rating of products provides a quick impression of the overall evaluation of the sex toy by the reviewer [10]. Furthermore, descriptions and narratives in a review help assess the technical characteristics of sex toys (e.g., size or smell), provide information related to the utilization of sex toys (e.g., use during partnered sex or solo sex), and perceived effects of their use (e.g., orgasm intensity). Online reviews reflect users’ experiences and might also help readers of reviews form impressions about sex toys [18,19].

### 1.2. Current Study

The aim of the current study is two-fold. First, we want to explore sex toy experiences that are publicly shared in online product reviews. Writing and reading public reviews of intimate products are, historically, fairly recent activities that did not exist in the pre-Internet era when sex toys were mainly marketed via mail order businesses [3].

Second, we are interested in the differences and similarities between the reviews of the different types of sex toys. Keeping in mind that sex toys come in different designs and shapes, we focused on realistic sex toys that resemble human bodies. We categorize realistic sex toys in a 2 × 2 design according to their representation of the human body (genitalia sex toys representing reproductive organs only versus torso toys representing larger parts of the human body) and their representation of gender (toys representing female versus male body parts). We talk about the gender of the sex toys because, as material artefacts, they do not have a biological sex for procreation. Instead their design is the result of a social construction of differently gendered and sexualized bodies.

The rationale behind this categorization is rooted in ongoing polarized debates about the benefits and risks of realistic and, hence, gendered sex toys, particularly in a heteronormative and patriarchal society [3]. On one side, sex toys (dildos and vibrators in particular) are endorsed by several feminist researchers and activists as empowering. In the context of heterosexual relations, vibrators can help women enjoy solo sexual activities and reach orgasms effortlessly and more independently from sexual partners, particularly from male sexual partners [20]. For heterosexual men, vibrators can also be liberating because they reduce performance pressure during partnered sex with women and can be used during solo sex regardless of sexual identity [21,22]. On another side, advanced sex toys such as torso sex toys, full-body sex dolls, or sex robots are argued to be dangerous because they invite *anthropomorphizing*: sex toys representing larger parts of the human body or the full human body might invite users to perceive and treat them like an artificial sexual partner [23]. Some feminist researchers and activists have raised concerns about female-bodied sex dolls and sex robots in particular because they allow men to objectify and abuse artificial women, a behavior that might foster the sexual objectification and abuse of real women [24,25].

These polarized feminist debates on sex toys addressing the empowerment and disempowerment of women are linked with sexual objectification theory [26] and sexual script theory [27].

In line with sexual objectification theory, realistic sex toys representing genitalia or larger parts of the human body can push traditional beauty norms [28]. Browsing through the *Sexual Wellness Products* section on Amazon.com at the time of data collection showed that most torso toys sold on the platform represent White, slim, young, non-disabled, cis-gender bodies. Research on full-body sex doll reviews and online forum discussions highlight that some male users indeed praise the super-natural beauty of their female dolls while complaining about the many flaws of real women [29]. Beauty norms can negatively affect men, women, and non-binary people alike, but researchers have demonstrated that women are particularly negatively impacted by strict beauty norms disseminated via mainstream media content and sexual product design [30,31].

The design of sex toys is not only relevant to appearance-related beauty standards but also to sexual behaviors. According to sexual script theory [27,32], the characteristics and functionalities of sex toys can shape their typical use. A common criticism of realistic sex toys is that they perpetuate the “coital imperative”, that is, their design (i.e., male and female genitalia) supports the idea that the main sexual activity and source of pleasure is penetration [33]. However, the feminist sex toy criticism is partly based on heteronormative assumptions, ignoring that different toys such as penis-shaped vibrators are also used by transgender people, during sex between women, and to penetrate heterosexual men [3]. Furthermore, the assumption that users of realistic female-bodied torso or full-body sex toys are driven by questionable (namely sexist, objectifying, and abusive) motives attaches stigma to the use of those toys [34].

Consumers also discuss sex toy usage with a focus on toy characteristics such as packaging, functionality, ease of use, and cleaning. Previous research on sex toy–related online reviews has pointed to both positive (“*the hottest toy ever*”) and negative (“*not in any way comparable to sex*”) usage experiences [9]. In a content analysis of *N* = 100 online sex toy reviews from popular magazines and websites, Rossolatos [35,36] found that reviewers’ experiences reflect both liberation through enhanced pleasure and “orgasms on the go” but also compliance to a logic of sexual efficiency in the sense that toys promise quick and easy orgasms.

The current study is the first to examine and compare online product reviews of four different types of realistic sex toys in a 2 × 2 design (see Table 1). The four research questions of the current study address evaluations of and experiences with the four types of sex toys. For each research question, we are interested in the general results across all toys combined as well as in the differences between the four types of toys.

The first research question (RQ) targets overall sex toy evaluations via star ratings:

RQ 1: What overall evaluation (star rating) do customers give to sex toys in Amazon product reviews, and do these evaluations differ between the four types of realistic toys?

Furthermore, we analyzed reviews containing information on (1) characteristics of the toys, (2) usage of the toys, and (3) perceived effects of the toys to answer the following research questions:

RQ 2: Which product characteristics of sex toys do customers mention in Amazon product reviews, and do these characteristics differ between the four types of realistic toys?

RQ 3: How do customers describe their use patterns of sex toys in Amazon product reviews, and do these use patterns differ between the four types of realistic toys?

RQ 4: How do customers describe the effects of their sex toy use in Amazon product reviews, and do these effects differ between the four types of realistic toys?

## 2. Materials and Methods

### 2.1. Study Design and Research Ethics

The current study employs a quantitative research approach and a 2 × 2 design incorporating four types of realistic sex toys, namely genitalia versus torso toys and male body versus female body toys (see Table 1). For data collection, we used media content analysis with online product reviews as the unit of analysis [37]. We followed an *open science approach*, that is, the codebook, data file, and data analysis scripts are publicly available via https://osf.io/b9dxa/.

Online reviews represent the opinions of a small group of sex toy users. However, the non-reactive data collection procedure allows us to collect data in a naturalistic setting: the Internet. Moreover, it is important to examine consumers’ preferences and attitudes toward sex toys given that their public online reviews can be influential on the opinions of potential consumers.

Using Amazon product reviews for the purpose of research appears to be ethically justified given that they are publicly accessible from any web-enabled device and do not require a user account to view them [38]. Previous empirical studies on Amazon product reviews share the same ethical position and consider the product reviews as public domain (e.g., [19,39]). To protect the privacy of the reviewers, we did not collect their usernames or any other identifying information.

### 2.2. Sampling and Data Collection

Data collection took place in October 2018 in the *Sexual Wellness Products* section on www.amazon.com. The website was accessed using the incognito mode of a web browser in order to avoid algorithmic patterns from previous user settings, preselected product suggestions, and biased search results. As the comparison groups were predefined by study design, we only assessed realistic genitalia and torso sex toys representing either male or female genders. For the group of genitalia sex toys, we selected the reviews from two best-selling and, hence, most reviewed products (one best-selling penis-shaped vibrator (G Spot Rabbit Vibrator with Bunny Ears for Clitoris Stimulation, PALOQUETH) and one best-selling vagina-shaped masturbator (Vibrating Male Masturbator Cup, PALOQUETH), resulting in *n* = 598 reviews. Torso sex toys had fewer reviews per product; thus, to balance out the total number of reviews, we collected reviews for this group from a total of nine best-selling torso toys (six male body and three female body toys), resulting in the group sample of *n* = 525. Amazon’s best sellers rank calculation is based on the number of sales within a product category and is updated hourly; therefore it can only be valid at the time of data collection.

Second, we applied the following inclusion criteria to the collected reviews: (1) the review contains enough information to be coded (i.e., more than a single word), (2) the review matches the selected product (i.e., it contains meaningful information related to the specific sex toy), and (3) the review is written in English. This resulted in a final quota sample of *N* = 778 Amazon product reviews suitable for further data analysis (see Table 1). After the pretest of the codebook, all collected reviews were coded by a single coder.

### 2.3. Instrument

#### 2.3.1. Codebook

The codebook was developed for the present study based on existing literature on sex toys and consumer research. We elaborated the codebook inductively by considering the specific characteristics of intimate products. The codebook contains 19 variables in four topic-specific blocks related to the four research questions (see Table 2). In addition, formal categories such as review title, date of publication, name and type of sex toy, as well as the coding date were included.

The first block related to RQ1 contains the star rating of sex toys as assigned by the reviewers. The second block focuses on product-related categories related to RQ2 and covers typical characteristics of durable consumer products derived from studies by Kotler et al. [40] and Archak et al. [16]. More specifically, the categories include assessment of a sex toy’s quality, size, weight, shape, smell, material, packaging, and pricing. The third block comprises categories related to the product’s use. First, it indicates the gender of the person who used the sex toy (men/women/both) and the type of sexual activity in which they used the sex toy (solo/partnered/both), both determined based on the review narrative. Second, it includes the utilization-related categories “functionality” and “ease of use” as derived from studies by Kotler et al. [40] and Archak et al. [16]. In addition, the category “cleaning” was included due to the prevalence of this topic in previous research on sexual products [41,42].

The fourth and final block of variables linked to RQ4 addresses categories related to the perceived positive and negative effects of sex toy use. These categories were created inductively from the collected Amazon reviews and deductively from studies on the negative and positive effects of sex toy use [21,43,44,45].

#### 2.3.2. Pretest

To pretest the codebook, two independent coders coded 100 randomly selected reviews. Overall, all variables showed good to almost perfect reliability with Gwet’s AC1 varying between 0.68 and 1.0 (see Table 2). We selected Gwet’s AC1 (first-order agreement coefficient) to calculate inter-coder reliability coefficients for nominal variables as it is typically less affected by prevalence and marginal probability than other measures of chance-corrected agreement [46]. Inter-coder reliability for star rating was measured with the intra-class correlation coefficient [47].

### 2.4. Data Analysis

We analyzed quantitative data using descriptive statistics (means and frequencies) and inferential statistics (*t*-tests, chi-square tests). The alpha level was set at 5%. Due to the exploratory character of the study, no corrections for cumulative Type I error rates were applied. All analyses were performed with R version 4.1.2 [48]. Descriptive statistics and *t*-tests were calculated with R base package, for chi-square tests and effect sizes packages gmodels [29] and rstatix [49] were used, respectively.

Additionally, we conducted a qualitative content analysis of the perceived positive and negative effects of sex toy use to get deeper insights into the consequences of sex toy use. Qualitative analysis followed an inductive thematic approach as it allows for data-driven analysis [50]. First, we collected all reviews explicitly addressing positive and/or negative effects of sex toy use on the user’s sexual health. Second, the reviews were examined in detail and short codes were assigned to each of them. The codes were then examined for common themes. Ultimately, all reviews were coded into identified themes. If a review mentioned multiple effects, multiple themes were coded. In addition to providing verbatim quotes, we also report descriptive statistics (frequencies) for the identified types of positive and negative effects. MS Excel Professional Plus 2019 was used for qualitative analysis.

## 3. Results

### 3.1. Sex Toy Evaluations

RQ1 assessed the overall star rating that customers gave to sex toys in their Amazon product reviews. Overall, consumers gave fairly high evaluations to the selected best-selling intimate products, with about 79% of reviews containing a four- or five-star rating. Across all sex toys, the vibrator received the highest star rating and torso sex toys the lowest (see descriptive results in Table 3). Group-wise, realistic genitalia sex toys received significantly higher ratings (*M* = 4.67, *SD* = 0.81) than torso toys (*M* = 3.62, *SD* = 1.50), *t* (532.27) = 11.83, *p* < 0.001, 95% *CI* [0.87, 1.22], *d* = 0.87. Male body–shaped toys received higher ratings (*M* = 4.36, *SD* = 1.28) than female body–shaped toys (*M* = 4.06, *SD* = 1.28), *t* (776) = 3.27, *p* =0.001, 95% *CI* [0.12, 0.49], *d* = 0.24.

### 3.2. Sex Toy Characteristics

RQ2 examined the product characteristics that were mentioned most frequently in Amazon product reviews of sex toys. The eight most common characteristics reviewers mentioned were: quality, size, weight, shape, smell, material, packaging, and price.

Across all comparison groups, product quality, material, and shape were addressed most frequently. The smell of the sex toy was the least mentioned characteristic. Regarding within-group differences, users of genitalia sex toys were more concerned with overall quality (*χ^2^* (1) = 38.53, *p* < 0.001, *V* = 0.22), material (*χ^2^* (1) = 9.06, *p* = 0.003, *V* = 0.10), and price (*χ^2^* (1) = 10.85, *p* < 0.001, *V* = 0.11) compared to users of torso toys. Users of torso toys addressed the characteristics size (*χ^2^* (1) = 93.66, *p* < 0.001, *V* = 0.34) and weight (*χ^2^* (1) = 65.35, *p* < 0.001, *V* = 0.29) more frequently than users of genitalia sex toys (see Table 4).

Within the gender comparison group, sex toy quality (*χ^2^* (1) = 8.67, *p* = 0.003, *V* = 0.10), size (*χ^2^* (1) = 19.65, *p* < 0.001, *V* = 0.16), and weight (*χ^2^* (1) = 16.16, *p* < 0.001, *V* = 0.14) were mentioned significantly more frequently in the reviews of female body toys compared to male body toys. In turn, reviews of male body toys addressed product packaging more frequently, *χ^2^* (1) = 12.57, *p* < 0.001, *V* = 0.12 (see Table 5).

### 3.3. Sex Toy Use

RQ3 assessed how Amazon reviewers described their use of sex toys. Overall, 54% of sex toy users represented in the product reviews were men, and 46% of sex toy users were women. Solo sex was the most common type of sexual activity in which the sex toy was used (69%) according to the analyzed reviews.

By analyzing the genders of the sex toy users represented in the reviews, we found that user gender significantly differed between genitalia versus torso sex toys, *χ^2^* (3) = 44.23, *p* < 0.001, *V* = 0.24 (see Table 6). Women represented in the product reviews were more likely to use genitalia toys as opposed to torso toys, and men were more likely to use torso toys as opposed to genitalia sex toys. Solo sexual activities were more common for torso toys, whereas using a sex toy with a partner was more typical for genitalia sex toys, *χ^2^* (3) = 11.30, *p* = 0.01, *V* = 0.12.

Regarding gender resemblances of sex toy use, women were more likely to use male body toys, whereas men were more likely to use female body toys, *χ^2^* (3) = 621.89, *p* < 0.001, *V* = 0.89 (see Table 7). The contexts in which sex toys were used differed significantly, *χ^2^* (3) = 106.43, *p* < 0.001, *V* = 0.37. Female body toys were mostly used in solo sexual activities, whereas male body toys were more frequently used during sex with a partner.

We examined three main topics of consumers’ use of sex toys: sex toy functionality, ease of use, and cleaning. In general, Amazon reviewers reported that sex toys exceeded their expectations regarding functionality, were easy to clean, and easy to use. All three topics were mentioned more often in reviews of genitalia sex toys than in reviews of torso sex toys (see Table 8). Reviewers of genitalia sex toys indicated that these types of sex toys function better than expected (59%), are easy to use (86%), and are easy to clean (76%). Reviewers found torso toys significantly more difficult to use compared to genitalia sex toys, *χ^2^* (1) = 49.43, *p* < 0.001, *V* = 0.65.

In the gender-resemblance comparison group, product functionality and ease of use were mentioned more often in reviews of male body toys than in reviews of female body toys (see Table 9). Cleaning was reported significantly more frequently in reviews of female body toys, *χ^2^* (1) = 25.05, *p* < 0.001, *V* = 0.18. Users of male body toys said that the toy exceeded their expectations regarding functionality more often than users of female body toys, *χ^2^* (2) = 27.64, *p* < 0.001, *V* = 0.23. Reviewers also found them significantly easier to use (*χ^2^* (1) = 9.41, *p* = 0.002, *V* = 0.29) and easier to clean (*χ^2^* (1) = 9.39, *p* = 0.002, *V* = 0.23).

### 3.4. Sex Toy Effects

For RQ4 the effects of sex toys mentioned in Amazon product review were investigated. We identified a total of *n* = 53 positive and *n* = 16 negative self-reported effects in our corpus of *N* = 778 reviews.

Positive effects were grouped into six main themes: (1) compensation for lack of a real-life sexual partner, (2) increased sexual pleasure, (3) addition to partnered sex, (4) improved mental health, (5) practice for future partnered sex, and (6) positive effects beyond sexual pleasure (see Table 10). Compensation for lack of a real-life sexual partner was the most reported effect. Reviewers considered sex toys both as a temporary replacement of an absent sexual partner (e.g., “*I am a widow now and currently do not have a gentleman friend so it comes in handy for me*”) and as a more permanent “better” solution to having one (e.g., “*It only costs money once, will never leave you and won’t mess with your head*”).

Negative effects were grouped into five main themes: (1) addictiveness, (2) inconvenience of use, (3) ineffectiveness, (4) problems with an existing partner, and (5) psychological tension (see Table 11). Almost half of the mentioned negative consequences involved the risk of getting addicted to a sex toy (44%) (e.g., “*The only thing that I worry about is the more I use it, the more I like it and I might not ever leave the house again*”).

## 4. Discussion

The aim of the current study was to explore consumers’ experiences with realistic sex toys via product reviews on Amazon.com. A total of *N* = 778 online reviews were collected in a 2 × 2 design covering genitalia versus torso sex toys as well as male versus female body sex toys.

We first assessed the overall evaluations (star ratings) consumers gave to best-selling realistic sex toys (RQ1). According to Amazon’s five-star rating system, most reviewers (79%) evaluated the best-selling sex toys from the selected categories positively by giving the products 4 or 5 stars. Consumers gave better ratings to genitalia sex toys compared to torso toys, and they gave better ratings to male body compared to female body toys. A star rating can indicate the overall valence of a review and therefore provide a quick overview of consumers’ experiences [22,24,28]. Thus, high star ratings of sex toys point to highly satisfactory user experiences with the respective intimate products. In particular, our results suggest that mainstream genitalia sex toys provided users with more satisfactory experiences than torso sex toys, and male body–shaped toys more satisfactory experiences than female body–shaped toys. Genital shape and anthropomorphism alone, of course, cannot fully explain the differences in the overall evaluations of selected sex toys. The quality of the specific products in the sample, their intended use, or differences in functionality between the toys of the same group are also expected to influence star ratings. Furthermore, it should be kept in mind that all sex toys in the sample were ranked as best sellers by Amazon. Although Amazon’s best seller rank is based on the number of sales only and not on the product evaluation, products in this category can be expected to receive overall higher star ratings.

We then examined the characteristics that consumers mentioned most frequently in their Amazon reviews of sex toys to get a deeper insight into the specific attributes of the sex toys (RQ2). Our results showed that general product quality, material, and shape were the most frequently mentioned characteristics across all types of sex toys. Reviewers were mainly concerned with sex toys’ overall quality such as their durability that is needed for smooth usage (e.g., “*Fantastic quality. Excellent density in structure. Will last for a very long time*”, “*Total crap. […] Broke after first usage*”). To be effective in generating or enhancing arousal, sex toys must be comfortable to use (e.g., a penis-shaped vibrator should not cause pain on insertion or distract a user by slipping out of their hands). Thus, reviewers paid a lot of attention to the material and shape of sex toys. Reviewers’ evaluations of the shape of sex toys entailed comments on their sexual appeal (“*Breasts are not 36DD as shown on picture*” [female torso toy]; “*At first glance I fell in love with the pretty and sleek design of this bad boy*” [vibrator]). In line with sexual objectification theory and previous empirical findings, this suggests that the design of the sex toys can push traditional beauty norms. It is especially visible in the reviews of female torso sex toys that were often commented on as having “too small”/“well rounded” breasts or a “curvy contour” of the waist. The lack of appearance diversity among torso sex toys contributes to the issue, although this claim is not supported by empirical data and is based on the observation during data collection.

Consumers of genitalia sex toys were significantly more concerned with quality and material compared to consumers of torso sex toys, while consumers of torso sex toys addressed size and weight more frequently. A possible explanation for this difference is that vibrators and masturbators have been on the market for a longer time than human torso sex toys and many consumers consider them to be mainstream products [4]. Therefore, their general functionality, look, and shape are already familiar to most people. Conversely, male and female torso sex toys are less widespread, considerably larger, and less portable than genitalia sex toys [28]. Not only does the storage of such toys require extra space, but their weight might pose an issue for some users (e.g., for people with disabilities). Furthermore, due to their larger dimensions, their ability to provide orgasms “on the go” [36] is limited. Hence, consumers addressed the size and the weight of torso sex toys as the most relevant factors in their reviews.

The users of male body toys addressed the packaging of sex toys two times more often than users of female body toys. It is assumed that statistically most users of male body toys are women [51]. Thus, women may address the appropriateness and discreteness of the packaging of sex toys more frequently given society’s stigmatized attitudes toward female users of intimate products. Nevertheless, this explanation is challenged by the small effect size of the difference we found as well as the overall low number of reviews that address this characteristic.

Regarding sex toy use (RQ3), our results showed that most sex toy users mentioned in the product reviews were men. While research has shown that sex toy use is somewhat more common in the female population [9], male users seem to write Amazon product reviews disproportionally more often. Our results showed that product reviewers predominantly reported about solo sexual activities but also mentioned partnered use of all four groups of toys. This observation adds to earlier research focusing on traditional vibrators only [20,21,22,44].

Three main utilization-related topics were investigated in the context of sex toy use: sex toys’ functionality, ease of use, and ease of cleaning. Users of both male and female torso sex toys reported torso sex toys to be difficult to use compared to a vibrator or a masturbator. This is most likely due to the naturally larger size and weight of torso sex toys that require some time to get accustomed to before comfortable use (e.g., “*there is no spine, makes it hard for it to be on top, flops around too much and when you pop out its annoying to go back in since you have to pick the whole thing up*”). Moreover, the general functionality of the selected best-selling torso sex toys was evaluated as worse than that of the selected best-selling genitalia toys. Many reviewers indicated that their expectations regarding functionality were not met. Combined with a more difficult use, results suggest that the use of torso sex toys was too complex and not intuitive enough for most reviewers, which can explain their overall lower star ratings (see Table 3).

Reviewers most frequently mentioned the topic of cleaning sex toys in reviews of female body toys as compared to male body toys. This finding is expected considering the general design of the product. Resemblance with female genitalia could be problematic for cleaning given that a vagina is bent inwards. Conversely, sex toys that resemble male bodies are usually shaped after an erect penis, so they can be cleaned much more easily. Certain risks associated with improper cleaning of sex toys (e.g., STI transmission) have been repeatedly raised in previous studies (e.g., [41,52]). However, a low number of reviews addressed the risks of improper sex toy cleaning in this study.

We also explored the perceived positive and negative effects of sex toy use (RQ4). In line with current research, consumers more frequently mentioned perceived positive effects of sex toys compared to negative effects. The *N* = 53 reviews that mentioned positive effects revealed that the use of sex toys helps to compensate for the lack of a sexual partner and to increase sexual pleasure (i.e., provide more intense and frequent orgasms). For genitalia sex toys, compensation for the lack of a real sexual partner typically meant that consumers could perform solo sexual activities in the temporary absence of a partner. For torso sex toys, some reviewers considered their toys as a complete replacement of a partner, or even as a better alternative to one (“*And not once have I heard him complain saying he’s tired nor that he has to go*”). This points to the anthropomorphization of those sex toys that are not limited to mere genitalia but represent larger parts of the human body and thus invite perceiving and treating the toy as an artificial partner [28]. The anthropomorphization of sex toys should, however, be evaluated with caution. In line with sexual objectification theory, earlier studies have already raised concerns that sexist, objectifying, and at times abusive use of human-like sex toys can lead to objectification and abuse of real-life sex partners, especially women (e.g., [24,25,34]). We have not found any evidence of physically abusive use of human-like sex toys in our sample; however, a number of reviews (predominantly reviews of torso sex toys) considered sex toys more appealing than a real partner for having no feelings, emotions, or complaints (“*Don’t worry about upset feelings. There are none away. It’s much easier and less trouble than a divorce or a nasty break up with a girlfriend. Just throw her out and away and get a nice new model to choose from on Amazon*”). Whether such opinions can impel objectification and emotional abuse of real-life partners present fruitful directions for future research.

Only *n* = 16 product reviews included perceived negative effects of sex toy use, with the most mentioned effect being the risk of getting addicted to the toy. Researchers have already highlighted addiction to sex toys as a concern [53]. However, the overall low number of negative reviews does not allow us to draw meaningful conclusions about the prevalence of this issue compared to other negative effects, such as the inconvenience of use or sex toys’ failure to enhance sexual sensations. Nevertheless, such low numbers of negative reviews could mean that people who leave product reviews perceive sex toy use as a mainly beneficial activity that has a positive impact on their life.

## 5. Limitations, Future Directions, and Implications

One of the main strengths of this study relies on the distinctions we made between different types of realistic sex toys. Earlier studies either had a single focus on mainstream products such as dildos or vibrators, or did not differentiate between different types of sex toys at all. However, the results of the study can only be considered as preliminary and further deeper investigation into the topic is needed. The collected product reviews refer to one best-selling, penis-shaped vibrator and one best-selling, vagina-shaped masturbator only, as well as to only a few best-selling torso sex toys. Hence, the results can be influenced both by the limited selection of products as well as their best-selling status. Future studies could cover reviews on more product examples for the four sex-toy types. In addition, a wider variety of intimate products should be investigated, such as remote-controlled vibrators and masturbators, torso toys with combined male and female genitalia (i.e., intersex), or full-body toys.

Furthermore, it should be noted that not all consumers leave product reviews. The reviews we analyzed in the present study might provide an initial introduction to the topic, but they only represent the opinions and experiences of a small proportion of sex toy users. Moreover, due to the anonymity of collected reviews, the sociodemographic characteristics of the reviewers are unknown. The conclusions about the genders of sex toy users were made based on the review narratives and the pronouns consumers used. It is not possible to know how the reviewers tested the sex toys before writing their evaluations (e.g., if they used the sex toys as indicated in the products’ user instructions).

Finally, the study is mostly based on research conducted on sex toy use in Western countries, where human sexuality is fairly liberated and openly discussed. In diverse cultural contexts, the results should be used with caution. An exploration of sex toy use in more conservative countries where sexuality is still considered a taboo topic and where religious norms may limit the openness of sexuality is recommended for future studies.

The product characteristics and effects of the sex toys addressed in product reviews might inspire product design and marketing. For example, providing and promising high-quality materials for all toys and ensuring easy handling of torso toys are key issues for customers. Sex educators and clinicians aiming to put sex toy use on the agenda can point their clients and patients to product reviews or use example reviews as conversation starters. Debating, developing, and exploring wholesome, diverse, and inclusive sex toys and use patterns remains an important task.

## 6. Conclusions

The current study contributes to and expands the existing research on sex toy use and perceived effects of usage by (1) focusing on actual user experiences as reflected in online product reviews and (2) comparing realistic genitalia sex toys with torso sex toys representing male and female bodies. The findings highlight that consumers openly share their intimate experiences with sex toys online and that these experiences vary depending on different types of products. This includes torso toys whose users are—together with users of full-body sex dolls—the subject of stigmatization and criticism by certain feminists. The public sharing of torso toy experiences, the partnered use of torso toys reported in 9% of the reviews, and also the predominantly positive perceived effects of their use could point to partial de-stigmatization. Furthermore, we found some indicators of people anthropomorphizing torso toys in product reviews. More specifically, some users saw the torso toy as an artificial partner and perceived this as a positive effect.

Over the last few decades, the online marketing of sex toys has presented more and more abstract designs of sex toys (e.g., vibrators elegantly shaped as silver cylinders) and predominantly framed sex toys as wellness products. Torso toys divert from this trend of minimalistic toys in their hyper-realistic, explicit, openly sexualized design (e.g., female torso toys with gaping labia and DD breasts). It is still unclear if torso toys, along with sex dolls and sex robots, will gather larger customer groups and contribute to sexual well-being as they become more visible and more openly discussed and evaluated on the Internet.

## Figures and Tables

**Table 1:** Quota sample of Amazon product reviews of four types of sex toys.

	Genitalia Sex Toys (*n* = 419)	Torso Sex Toys (*n* = 359)
**Male body sex toys** **(*n* = 329)**	Vibrator (*n* = 226)	Male torso sex toy (*n* = 103)
**Female body sex toys** **(*n* = 449)**	Masturbator (*n* = 193)	Female torso sex toy (*n* = 256)

Note. *N* = 778. For visualizations of the toys addressed by the online reviews.

**Table 2:** Inter-coder reliability of the 19 codebook variables related to the 4 research questions.

Category	Gwet’s AC1	Agreement %
**Category for RQ1**		
Star rating	1.0 ^a^	100 ^a^
**Categories for RQ2**		
Sex toy quality	0.70	78
Sex toy size	0.88	93
Sex toy weight	0.94	95
Sex toy shape	0.68	82
Sex toy smell	0.98	98
Sex toy material	0.82	91
Sex toy packaging	1.0	100
Sex toy price	0.94	95
**Categories for RQ3**		
User gender	0.77	81
Type of sexual activity	0.71	76
Functionality of the sex toy	0.71	84
Functionality evaluation	0.66	74
Ease of use of sex toy	0.72	80
Ease of use evaluation	0.77	79
Cleaning of sex toy	0.95	97
Cleaning evaluation	0.94	95
**Categories for RQ4**		
Positive effects	0.92	93
Negative effects	0.88	90

Note. ^a^ Star rating as a metric variable simply to be copied from Amazon to the data file reached a reliability coefficient of ICC = 1.0.

**Table 3:** Average star ratings of sex toys in Amazon product reviews.

Product Type	*n*	*%*	Star Rating	
			*M*	*SD*
Vibrator	226	29	4.83	0.60
Masturbator	193	25	4.48	0.97
Male torso	103	13	3.34	1.71
Female torso	256	33	3.74	1.38
**Total**	**778**	**100**	**4.19**	**1.29**

Note. Amazon’s star ratings follow a five-star system with one star being the lowest rating and five stars being the highest rating.

**Table 4:** Frequency of mentioned product characteristics of genitalia versus torso sex toys in Amazon product reviews.

Product Characteristic	Total (*N* = 778)		Genitalia Sex Toys (*n* = 419)		Torso Sex Toys (*n* = 359)		*χ^2^* (1)	*p*	*V*
	*n*	*%*	*n*	*%*	*n*	*%*			
Quality	373	48	244	63	129	36	38.53	<0.001	0.22
Material	366	47	218	52	148	41	9.06	0.003	0.10
Shape	335	43	165	39	170	47	5.01	0.025	0.08
Size	229	29	62	15	167	47	93.66	<0.001	0.34
Price	190	24	122	29	68	19	10.85	<0.001	0.11
Packaging	113	15	86	21	27	8	26.34	<0.001	0.18
Weight	83	11	10	2	73	20	65.35	<0.001	0.29
Smell	50	6	18	4	32	9	6.85	0.009	0.09

Note. The characteristics of the sex toys are presented in descending order based on the frequency of their being mentioned in online reviews.

**Table 5:** Frequency of mentioned product characteristics of male versus female body toys in Amazon product reviews.

Product Characteristic	Total (*N* = 778)		Male Body Sex Toys (*n* = 329)		Female Body Sex Toys (*n* = 449)		*χ^2^ (1)*	*p*	*V*
	*n*	*%*	*n*	*%*	*n*	*%*			
Quality	373	48	178	54	195	43	8.67	0.003	0.10
Material	366	47	148	45	218	49	0.97	0.325	0.03
Shape	335	43	129	39	206	46	3.44	0.063	0.06
Size	229	29	69	21	160	36	19.65	<0.001	0.16
Price	190	24	69	21	121	27	3.67	0.055	0.07
Packaging	113	15	65	20	48	11	12.57	<0.001	0.12
Weight	83	11	18	5	65	14	16.16	<0.001	0.14
Smell	50	6	27	8	23	5	3.00	0.083	0.06

Note. The characteristics of the sex toys are presented in descending order based on the frequency of their being mentioned in online reviews.

**Table 6:** Frequency of mentioned user gender and type of sexual activity for genitalia versus torso sex toys in Amazon product reviews.

	Total (*N* = 778)		Genitalia Sex Toys (*n* = 419)		Torso Sex Toys (*n* = 359)		*χ^2^ (3)*	*p*	*V*
	*n*	*%*	*n*	*%*	*n*	*%*			
**User gender**									
Women	219	28	154	37	65	18	44.23	<0.001	0.24
Men	423	54	185	44	238	66			
Both	12	2	5	1	7	2			
Unknown	124	16	75	18	49	14			
**Sexual activity**									
Solo sex	534	69	270	64	264	74	11.30	0.01	0.12
Partnered sex	84	11	52	12	32	9			
Both	33	4	25	6	8	2			
Unknown	127	16	72	17	55	15			

Note. Percentages may not add up to 100 due to rounding.

**Table 7:** Frequency of mentioned user gender and type of sexual activity for genitalia versus torso sex toys in Amazon product reviews.

	Total (*N* = 778)		Male Body Sex Toys (*n* = 329)		Female Body Sex Toys (*n* = 449)		*χ^2^ (3)*	*p*	*V*
	*n*	*%*	*n*	*%*	*n*	*%*			
**User gender**									
Women	219	28	215	65	4	1	621.89	<0.001	0.89
Men	423	54	10	3	413	92			
Both	12	2	10	3	2	0			
Unknown	124	16	94	29	30	7			
**Sexual activity**									
Solo sex	534	69	162	49	372	83	106.43	<0.001	0.37
Partnered sex	84	11	67	20	17	4			
Both	33	4	20	6	13	3			
Unknown	127	16	80	24	47	11			

Note. Percentages may not add up to 100 due to rounding.

**Table 8:** Frequency of mentioned utilization-related topics for genitalia versus torso sex toys in Amazon product reviews.

Topic	Total (*N* = 778)		Genitalia Sex Toys (*n* = 419)		Torso Sex Toys (*n* = 359)		*χ^2^*	*df*	*p*	*V*
	*n*	%	*n*	%	*n*	%				
**Functionality mentioned**	517	66	325	78	192	53	50.30	1	<0.001	0.25
Functionality			325	100	192	100	37.81	2	<0.001	0.27
Better than expected	262	51	191	59	71	37				
As expected	197	38	116	36	81	42				
Worse than expected	58	11	18	6	40	21				
**Cleaning mentioned**	155	20	102	24	53	15	11.12	1	0.001	0.12
Cleaning			102	100	53	100	13.43	1	<0.001	0.28
Easy to clean	103	66	78	76	25	47				
Not easy to clean	52	34	24	24	28	53				
**Ease of use mentioned**	111	14	73	17	38	11	7.39	1	0.007	0.09
Ease of use			73	100	38	100	49.43	1	<0.001	0.65
Easy to use	70	63	63	86	7	18				
Not easy to use	41	37	10	14	31	82				

**Table 9:** Frequency of mentioned utilization-related topics for male versus female body toys in Amazon product reviews.

Topic	Total (*N* = 778)		Male Body Sex Toys (*n* = 329)		Female Body Sex Toys (*n* = 449)		*χ^2^*	*df*	*p*	*V*
	*n*	%	*n*	%	*n*	%				
**Functionality mentioned**	517	66	237	72	280	62	7.97	1	0.005	0.10
Functionality			237	100	280	100	27.64	2	<0.001	0.23
Better than expected	262	51	147	62	115	41				
As expected	197	38	62	26	135	48				
Worse than expected	58	11	28	12	30	11				
**Cleaning mentioned**	155	20	38	12	117	26	25.05	1	<0.001	0.18
Cleaning			38	100	117	100	9.39	1	0.002	0.23
Easy to clean	103	66	33	87	70	60				
Not easy to clean	52	34	5	13	47	40				
**Ease of use mentioned**	111	14	48	15	63	14	0.05	1	0.826	0.004
Ease of use			48	100	63	100	9.41	1	0.002	0.27
Easy to use	70	63	38	79	32	51				
Not easy to use	41	37	10	21	31	49				

**Table 10:** Frequency of mentioned positive effects of sex toys in Amazon product reviews.

Theme	Example	*n*	*%*
Compensation for lack of a real-life sexual partner	“I am a widow now and currently do not have a gentleman friend so it comes in handy for me” (review of a vibrator) “This is a good time!!! I dress her up, talk to her, and give her what she needs! No drama, no STDs.” (review of a female torso sex toy)	20	38
Increased sexual pleasure	“Using this gives me some of the most intense orgasms I’ve ever had!” (review of a masturbator) “The 7–8 inch penis always stays hard so when you’re ready to cum the penis stays hard which gives me a harder more intensive orgasm” (review of a male torso sex toy)	13	25
Addition to partnered sex	“With my new magical friend, we can both get what we need and it has brought our sexual relationship to a whole new level and our intimacy is much closer and deeper” (review of a vibrator) “It definitely added fun while playing because it makes you feel like three people have sex together” (review of a male torso sex toy)	11	21
Improved mental health	“Definitely something to consider if true inner peace is something that interests you” (review of a masturbator) “Helped me get over my ex” (review of a female torso sex toy)	4	8
Practice for future partnered sex	“Definitely good practice for the real thing” (review of a masturbator) “It makes you work on your technique for the actual thing. If you get some” (review of a female torso sex toy)	3	6
Positive effects beyond sexual pleasure	“Its pretty good when you’re bored or having trouble sleeping for sure!” (review of a masturbator) “I’ve been knocking boots with it twice a day and my abs are starting to show again. lol its a great workout.” (review of a male torso sex toy)	2	4

Note. *N* = 53 Amazon reviews that address perceived positive effects of sex toys. Percentages may not add up to 100 due to rounding.

**Table 11:** Frequency of mentioned negative effects of sex toys in Amazon product reviews.

Theme	Example	*n*	*%*
Addictiveness	“The only thing that I worry about is the more I use it, the more I like it and I might not ever leave the house again…” (review of a vibrator) “Now I can’t sleep without this every night!” (review of a male torso sex toy)	7	44
Inconvenience of use	“I am a little older so a good hard erection is not as easy anymore. So that is the only issue I have had is being able to get inside the toy.” (review of a masturbator) “It is hard for me to cum with the dick bending and making a cracking sound. It is uncomfortable and loud. I almost woke my parents up” (review of a male torso sex toy)	4	25
Ineffectiveness	“Does not provide any more intense of an orgasm than using perhaps a homemade device, to be quite frank” (review of a female torso sex toy) “Have to use other things this doesn’t really help” (review of a female torso sex toy)	3	19
Problems with an existing partner	“Girlfriend doesn’t need me anymore” (review of a vibrator)	1	6
Psychological tension	“it felt like I was holding a baby, which made the sex very uncomfortable. I’m definitely not interested in being a pedophile” (reviews of a female torso sex toy)	1	6

Note. *N* = 16 Amazon reviews that address perceived negative effects of sex toys. Percentages may not add up to 100 due to rounding.

## Data Availability

The study follows an open science approach, that is, the codebook, data file, and data analysis scripts are publicly available via https://osf.io/b9dxa/ (accessed on 13 April 2022).
